# Brain structure in women at risk of postpartum psychosis: an MRI study

**DOI:** 10.1038/s41398-017-0003-8

**Published:** 2017-12-18

**Authors:** Montserrat Fusté, Astrid Pauls, Amanda Worker, Antje A. T. S Reinders, Andrew Simmons, Steven C. R. Williams, Josep M. Haro, Kate Hazelgrove, Susan Pawlby, Susan Conroy, Costanza Vecchio, Gertrude Seneviratne, Carmine M. Pariante, Mitul A. Mehta, Paola Dazzan

**Affiliations:** 1 Department of Psychosis Studies, Institute of Psychiatry, Psychology & Neurosciences, King’s College London, De Crespigny Park, London, UK SE5 8AF; 2 CIBERSAM, Centro de Investigación Biomédica en Red de Salud Mental, Madrid, Spain; 3 Department of Neuroimaging, Institute of Psychiatry, Psychology & Neurosciences, King’s College of London, De Crespigny Park, London, UK; 4 National Institute for Health Research (NIHR) Mental Health Biomedical Research Centre at South London and Maudsley NHS Foundation Trust and King’s College London, London, UK; 5 Section of Stress, Psychiatry and Immunology and Perinatal Psychiatry, Department of Psychological Medicine, Institute of Psychiatry, Psychology & Neurosciences, King’s College London, London, UK

## Abstract

Postpartum psychosis (PP) is the most severe psychiatric disorder associated with childbirth. The risk of PP is very high in women with a history of bipolar affective disorder or schizoaffective disorder. However, the neurobiological basis of PP remains poorly understood and no study has evaluated brain structure in women at risk of, or with, PP. We performed a cross-sectional study of 256 women at risk of PP and 21 healthy controls (HC) in the same postpartum period. Among women at risk, 11 who developed a recent episode of PP (PPE) (*n* = 2 with lifetime bipolar disorder; *n* = 9 psychotic disorder not otherwise specified) and 15 at risk women who did not develop an episode of PP (NPPE) (*n* = 10 with lifetime bipolar disorder; *n* = 1 with schizoaffective disorder; *n* = 1 with a history of PP in first-degree family member; *n* = 3 with previous PP). We obtained T1-weighted MRI scans at 3T and examined regional gray matter volumes with voxel-based morphometry and cortical thickness and surface area with Freesurfer. Women with PPE showed smaller anterior cingulate gyrus, superior temporal gyrus and parahippocampal gyrus compared to NPPE women. These regions also showed decreased surface area. Moreover, the NPPE group showed a larger superior and inferior frontal gyrus volume than the HC. These results should be interpreted with caution, as there were between-group differences in terms of duration of illness and interval between delivery and MRI acquisition. Nevertheless, these are the first findings to suggest that MRI can provide information on brain morphology that characterize those women at risk of PP more likely to develop an episode after childbirth.

## Introduction

Puerperal (or postpartum) psychosis (PP) typically occurs within the first 6 weeks after delivery. PP is the most severe psychiatric disorder associated with childbirth^1^, and is highly predictable: the risk has been reported to range between 25 and 50% in women with schizoaffective disorder or bipolar affective disorder^2–4^ and up to 50–70% in women with a previous history of PP^5^ or both bipolar disorder and family history of PP.^3^

Most studies conducted to date in women at risk of, or with PP, have focused on clinical presentation, treatment or genetic factors. In contrast, there have been no studies on brain structure in these women. This is surprising considering that structural magnetic resonance imaging (MRI) has been extensively used to study the brain in patients with affective and non-affective psychosis unrelated to the puerperium, and in individuals at risk of developing a psychotic disorder because of genetic or clinical factors.

Only one study has evaluated brain volumes in women with PP, using computerized tomography and reporting an enlargement of ventricular and superior cerebellar cistern volumes in women with PP compared to women with bipolar disorder or other psychotic illness and healthy women^6^. More recent studies have used MRI in individuals with, or at risk of, psychoses unrelated to the puerperium. Studies in patients with bipolar disorder have reported smaller volumes of the anterior cingulate cortex (ACC)^7–9^ and of paralimbic regions, areas implicated in emotional processing^10–12^, in affected individuals. Moreover, when evaluating subtypes of bipolar affective disorder, patients with bipolar disorder I and bipolar disorder II displayed not only reductions in volume but also a decrease in cortical thickness and surface area in frontal brain regions relative to healthy controls (HC). Importantly, they differed in temporal and medial prefrontal regions, where only bipolar disorder I patients showed lower cortical volume and thickness, but not surface area abnormalities, suggesting diagnosis-related cortical difference^13^.

Studies in patients with schizophrenia and other non-affective psychoses have found more extensive gray matter reductions in frontal, limbic and neocortical structures, including hippocampus and superior temporal gyrus (STG) as well as larger ventricular volumes^14–16^. Work from our group and others have shown that some of these alterations are already present in individuals without psychosis but at high risk of developing an episode. These include reductions in volume in the hippocampus, STG, insula and cingulate gyrus^17, 18^. At risk individuals who convert to psychosis have also shown steeper rates of thinning in prefrontal cortex^19^. Furthermore, some of these changes seem specific to the subsequent type of psychosis, with those individuals who later develop an affective psychosis showing smaller subgenual cingulate volume, a region implicated in emotional processing and in generating affective symptoms^20^^,^^21^, than those who develop schizophrenia^22^^,^^23^. Interestingly, this region has also been found to be smaller in other at risk groups, such as first-degree relatives of patients with bipolar disorder^24^. Likewise, a recent study evaluated cortical thickness (CTh) and cortical surface area (CSA) in the offspring of patients with schizophrenia and of patients with bipolar disorder^25^. The authors found that the offspring of patients with schizophrenia had smaller global, parietal and occipital lobe surface area compared with the offspring of HC, and also of the occipital lobe compared with the offspring of patients with bipolar disorder. There were however no differences between the groups in cortical thickness.

Relevant to our study, PP remains debated as a diagnostic entity, possibly because of its polymorphic clinical symptoms, its various risk factors and the abrupt onset occurring when hormonal changes take place^26^. Since its clinical presentation shares similarity with that of affective psychoses, and considering the risk is higher in those women with bipolar disorder, it could be hypothesized that brain structural abnormalities in women at risk of developing PP would overlap, at least in part, with those of individuals with a vulnerability for, or an established, affective psychosis.

Here, we used MRI for the first time to examine whether women who develop a PP episode (PPE) differ in brain cortical volume and morphology from women who are at risk of PP but do not develop PP episodes (NPPE), and from women without mental illness in the same postpartum period. We estimated voxel-based morphometry and surface-based cortical volumes. Cortical volumes are the product of CSA and CTh, which represent distinct aspects of cortical architecture and could follow different developmental trajectories^27^. Using both approaches allows an evaluation of whether differences in cortical morphology, such as regionally increased or decreased thickness or area, are consistent with those observed with voxel-based morphometry, providing further insight into any observed morphological change.

We predicted that: (1) compared to healthy women, those at risk of developing PP would present an imaging profile similar to that observed in both affective psychosis and non-affective psychoses—primarily volume reductions in frontal and temporal regions; and (2) those at risk women who developed PP would present reductions of areas particularly involved in affective psychoses, such as ACC and hippocampus. In addition, we explored differences in CTh and CSA measures in those regions with cortical volume changes.

## Methods and materials

### Sample

Women at risk of PP were recruited from perinatal psychiatric services in London. Women were considered at risk if they had a diagnosis of bipolar disorder, schizoaffective disorder, had suffered a previous episode of PP or had a history of PP in a first-degree relative. Women were identified in pregnancy or within the first year postpartum and were scanned within 10 months from childbirth. Women gave written consent and the study was approved by the local Research Ethics Committee (10/H0807/14).

We recruited 26 women at risk: *n* = 11 had a recent episode of PP (PPE) and *n* = 15 had not developed an episode of PP in the postpartum period (NPPE). In the PPE group, *n* = 2 women had a DSM-IV diagnosis of bipolar disorder; *n* = 5 psychotic disorder not otherwise specified; *n* = 4 first PPE. In the NPPE group, *n* = 10 had bipolar disorder; *n* = 1 schizoaffective disorder; *n* = 1 first-degree family history of PP; and *n* = 3 PP in previous pregnancy but not in the most recent pregnancy. We also recruited *n* = 21 HC from local obstetric services, matched to the at risk group according to age, parity and ethnicity. Inclusion criteria for all women were: age 18–45 years, ability to communicate in English, no severe obstetric complications in most recent pregnancy; no MRI contraindications. Additional inclusion criteria for the HC were no personal history of bipolar disorder, schizophrenia or schizoaffective disorder, no personal or family history of PP and no medication at the time of assessment. Sociodemographic and clinical data are shown in Table 1. Women in the PPE group had a later age of onset (27 ± 6.4 years vs 22 ± 6.4; *p* = 0.01), shorter duration of illness (3.9 ± 5.2 vs 10.9 ± 6.8 years; *p* = 0.01) and the MRI scan considering weeks after delivery was performed later (24.8 ± 13 vs 11 ± 4 weeks) than women in the NPPE group.

**Table 1:** Sociodemographic and clinical data

	HC (*n* = 21)	Women at risk (*n* = 26)		
		NPPE (*n* = 15)	PPE (n = 11)	Statistic (df, *N*)
Sociodemographic variables				
Age (mean years ± SD)	35.4 ± 4.4	32.8 ± 5.4	32.4 ± 3.5	F (2, 47) = 2.08; *p* = 0.136
Ethnicity (Caucasian %; *n*)	76% (16)	60% (9)	45.5% (5)	*χ* ^2^ (2, 47) = 3.09; *p* = 0.211
Weeks after delivery ( ± SD)	14.4 ± 10	12.3 ± 6	24.8 ± 13	F (2, 47) = 4.2; *p* = 0.02
Parity (primiparity %; *n*)	67% (14)	47% (7)	70% (7)	*χ* ^2^ (6, 46) = 4.94; *p* = 0.6
Right Handedness (%; *n*)	85% (17)	100% (12)	91% (10)	*χ* ^2^ (2, 46) = 2.43*; p* = 0.29
Clinical characteristics				
Age of onset (mean years ± SD)	N/A	22 ± 6.4	27.8 ± 3.9	*t* = −2.68; *p* = 0.01
Time between MRI and onset illness (years)	N/A	10.9 ± 6.8	3.9 ± 5.2	*t* = 2.78; *p* = 0.01
Medication (yes %; *n*)	N/A	50% (5)	75% (9)	*χ* ^2^ (2, 26) = 3.56;* p* = 0.16
Antipsychotic daily dose (mg chlorpromazine equivalents)	N/A	285.2 ± 231	359 ± 243	*t* = −0.55; *p* = 0.59
PANSS Total score	31 ± 2.0	37 ± 7.9	40 ± 6.6	*K* (2, 47) = 18,4; *p* < 0.001
YMRS Total score	0.5 ± 0.8	1.6 ± 2.4	2.1 ± 1.4	*K* (2, 47) = 10,5;* p* = 0.002
HDRS Total score	2.1 ± 3.0	7.3 ± 6.3	6.5 ± 4.6	*K* (2, 47) = 13,6;* p* = 0.004

Note: *HDRS* Hamilton Depression Rating Scale*, MRI* magnetic resonance imaging, *PANSS* Positive and Negative Syndrome Scale, *YMRS* Young Mania Rating Scale. Post-hoc analysis for weeks after delivery showed the differences were among the NPPE and PPE groups (*p* = 0.028). Post-hoc analyses for the total PANSS Score, YMRS and HDRS showed no differences between the NPPE and PPE groups (*p* = 0.287), (*p* = 1.000) and (*p* = 0.894), respectively

### Clinical assessments

Current and Lifetime diagnoses were evaluated with the Structural Clinical Interview for the Diagnostic and Statistical Manual of Mental Disorders, Fourth Edition (SCID) (DSM-IV)^25, 28^. Symptom severity was assessed with the Positive and Negative Syndrome Scale (PANSS)^29^, the Young Mania Rating Scale (YMRS)^30^ and the Hamilton Depression Rating Scale (HDRS)^31^. The dose of antipsychotic medications on the day of the scanning session was converted into chlorpromazine equivalents^32^.

### Structural MRI

Women were scanned on a General Electric Signa HDx 3 Tesla scanner at the Centre for Neuroimaging Sciences, South London and Maudsley NHS Foundation Trust, with the body coil used for RF transmission and an eight-channel head coil for RF reception. A high-resolution T1-weighted inversion recovery prepared SPGR scan was acquired (TR = 6.988 ms, TE = 2.812 ms, TI = 400 ms 196 coronal slices, voxel dimensions 1.09 × 1.09 × 1.1 mm, matrix size 256 × 256).

### Image analysis

#### Voxel-based morphometry

Imaging data were transferred and processed on a Microsoft Windows platform using MATLAB R2008b (The MathWorks Inc., Natick, MA, USA) and Statistical Parametric Mapping software (SPM8; The Wellcome Department of Imaging Neuroscience, London, UK; see www.fil.ion.ucl.ac.uk/spm). Following inspection for image artefacts,^33^ T1-weighted images were aligned manually along the Anterior–Posterior Commissure followed by segmentation to extract the data into gray matter, white matter and cerebrospinal fluid compartments. A Diffeomorphic Anatomical Registration using Exponentiated Lie algebra (DARTEL) segmentation was implemented. Resulting gray matter images were smoothed with a 10mm isotropic Gaussian kernel in order to compensate for the inexact nature of spatial normalization and to maximize the chances that regional effects are expressed at a spatial scale where homologies in structural anatomy exist over subjects.

#### Cortical thickness and Cortical surface area

Surface-based analyses were performed using FreeSurfer version 5.1.0 (Massachusetts General Hospital, Harvard Medical School; http://surfer.nmr.mgh.harvard.edu). This includes removal of non-brain tissue^34^, Talairach transformation, segmentation of subcortical structures^35^, intensity normalization^36^, tessellation of GM/WM boundary, automated topology correction^34^ and surface deformation by following the intensity gradients to demarcate the GM/WM and GM/cerebrospinal fluid borders at the locations where the greatest shift intensity occurred^37^. The cerebral cortex is then parcellated into units based on gyral and sulcal structure allowing local curvature and CSA measures to be computed.

### Statistical analysis

We estimated between-group differences for continuous variables using one-way ANOVA or *t*-test as appropriate, and Mann–Whitney, Kruskal–Wallis or Chi-square tests for non-parametric data. Post-hoc analyses were carried out when the three-group comparison was statistically significant. Significance was set at a *p*-value <0.05; all *p*-values were two-tailed. Statistical analyses of demographic and clinical data were performed with the Statistical Package for the Social Sciences (SPSS version 21).

Brain-wide between-group comparisons were performed voxel-wise following the principles of the general linear model, with total GM Volume (TGMV) as nuisance covariate. We first performed a voxel-wise comparisons between PPE, NPPE and HC, using ANOVA, and a two sample *t*-test to directly compare the two experimental subgroups, NPPE and PPE. In addition, we performed a series of region of interest analyses. More specifically, we created anatomical masks of anterior cingulate gyrus (ACC), each hippocampus and parahippocampal gyrus, bilateral STG, superior and inferior gyrus and postcentral gyrus, with the WFU PickAtlas toolbox using the AAL atlas^38^. We subsequently applied Small Volume Correction procedures and age as a covariate. Significance level was set at *p* < 0.05 after family-wise error correction for multiple comparisons. We conducted CTh and CSA analyses using the Desikan atlas^39^ to further explore the between-group differences identified by the voxel-based morphometry analysis. We ran a general linear model including age as covariate for each region, reporting CTh and CSA values separately.

Our analyses examined voxel-wise volumetric differences between PPE and NPPE women, and HC; and then explored volume differences in hippocampi and parahippocampal gyrus, anterior cingulate gyrus, STG, postcentral, superior and inferior frontal gyrus, as areas affected in individuals at risk of psychotic spectrum disorders. Finally, we assessed CTh and CSA of regions of interest that significantly differed between groups.

## Results

### Voxel-based morphometry analysis

Voxel-wise comparisons were initially restricted to our a priori regions of interest: ACC, postcentral gyrus, bilateral inferior and frontal gyrus, STG, hippocampus and parahippocampal gyrus.

**Fig. 1:**
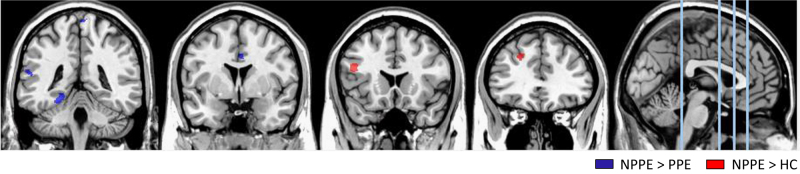
Brain coronal sections showing contrasts between HC, NPPE and PPE. Blue: smaller left parahippocampal gyrus, superior temporal gyrus, postcentral gyrus and anterior cingulate cortex volume in relation to PPE; red: larger inferior frontal gyrus and superior frontal gyrus volume in NPPE women than in HC after applying SVC–FWW *p* < 0.05

Women with PPE, compared to NPPE women, had smaller volume of ACC, left parahippocampal gyrus, left STG, and at a trend level postcentral gyrus (Table 2 and Fig. 1). There were no differences between women with PPE and HC in these regions.

**Table 2:** Differences in gray matter volume of regions of interest between women with postpartum psychosis episode (PPE), women with No Postpartum Psychosis Episode (NPPE) and Healthy Controls (HC), after applying Small Volume Correction

Cortical region	MNI coordinates (*x*, *y*, *z*)	Size in voxels	Z-score	*p*-value
A priori MRI ROI analysis				
NPPE > PPE				
Anterior cingulate cortex	4, −1, 36	94	3.34	0.040 after FWE correction
Left parahippocampal gyrus	−21, −37, −12	105	3.61	0.017 after FWE correction
Left superior temporal gyrus	−57, −34, 15	293	3.56	0.017 after FWE correction
Postcentral gyrus	6, −39, 78	20	3.43	0.076 after FWE correction
Left superior frontal gyrus	−26, 29, 34	20	3.04	0.070 after FEW correction
Left inferior frontal gyrus	−44, 35, 13	163	3.43	0.061 after FEW correction
NPPE > HC				
Left superior frontal gyrus	−26, 29, 34	92	4.09	0.010 after FWE correction
Left inferior frontal gyrus	−45, 17, 19	124	4.03	0.007 after FWE correction

Note: One patient was excluded in NPPE group (*n* = 14) due to different acquisition parameters for the analysis

In contrast, women with NPPE had larger left superior and inferior frontal gyrus than HC and, at trend level, also of PPE (Table 2 and Fig. 1). Differences between the NPPE and PPE groups remained even when we covaried for daily antipsychotic dose, PANSS, YMRS and HDRS symptom severity.

Whole brain voxel brain-wise comparison of gray matter volumes showed no differences between PPE, NPPE and HC after applying family-wise error correction for multiple comparisons.

### Cortical thickness and surface area region of interest analysis

**Fig. 2:**
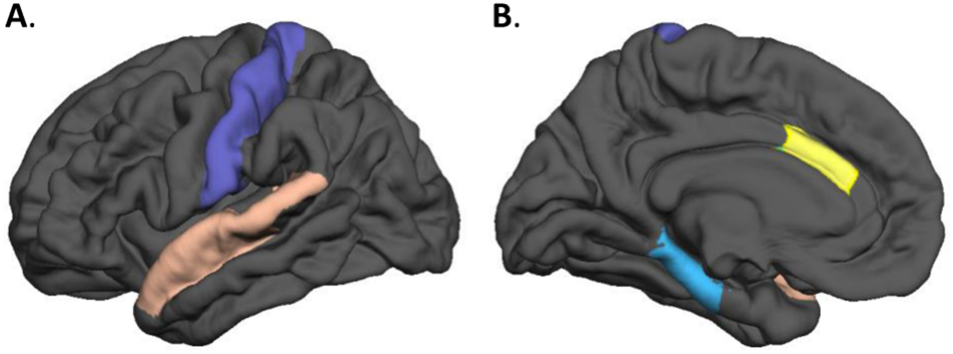
Illustration representing the four regions that showed smaller areas in PPE compared to NPPE. (**a**) Postcentral gyrus in purple and ACC in yellow. (**b**) Parahippocampal gyrus in blue and superior temporal gyrus (STG) in pink

We subsequently investigated CTh and CSA in the regions that differed in volume between groups. Women with PPE showed smaller CSA than women with NPPE in left parahippocampal gyrus, ACC, postcentral gyrus and left STG. However, the post-hoc analysis in left STG and postcentral area did not show significant differences between the groups (Table 3 and Fig. 2). We did not find any significant difference in CTh for any of these areas. There were also no significant differences between NPPE and HC in either CTh or CSA of superior or inferior frontal gyrus.

**Table 3:** Differences in cortical thickness (CTh) and cortical surface area (CSA)

CTh	HC	NPPE	PPE	Statistics (df, *N*)
Caudal anterior cingulate gyrus	2.6 ± 0.35	2.6 ± 0.22	2.7 ± 0.31	F(2, 46) = 0.29; *p* = 0.74
Left parahippocampal gyrus	3.2 ± 0.28	3.1 ± 0.25	3.3 ± 0.30	F(2, 46) = 1.17; *p* = 0.188
Left superior temporal gyrus	2.7 ± 0.18	2.9 ± 0.15	2.7 ± 0.11	F(2, 46) = 1.81; *p* = 0.175
Postcentral gyrus	2.2 ± 0.16	2.1 ± 0.15	2.2 ± 0.16	F(2, 46) = 1.12; *p* = 0.33
Left inferior frontal gyrus	2.2 ± 0.16	2.2 ± 0.13	2.2 ± 0.16	F(2, 46) = 0.56; *p* = 0.94
Left superior frontal gyrus	2.8 ± 0.17	2.9 ± 0.15	2.8 ± 0.11	F(2, 46) = 1.68; *p* = 0.19
CSA	HC	NPPE	PPE	Statistics (df, *N*)
Caudal anterior cingulate area	689.6 ± 155.5	693.7 ± 192.8	539.8 ± 90.2	F(2, 46) = 3.91; *p* = *0.04*
Left parahippocampal area	673 ± 79.7	791.5 ± 164.7	672.4 ± 136.7	F(2, 46) = 4.33; *p* = 0.01
Left superior temporal area	3288 ± 363.9	3514.3 ± 455.1	3139.1 ± 354.8	F(2, 46) = 3.19; *p* = 0.05
Postcentral area	3749.4 ± 395.7	3861.5 ± 400.6	3417.1 ± 477.8	F (2, 46) = 3.63; *p* = 0.04
Left inferior frontal area	3853.9 ± 555.7	4147.1 ± 440.7	3927.6 ± 454.1	F (2, 46) = 2.06; *p* = 0.13
Left superior frontal area	6361. ± 713.5	6695.1 ± 863.4	6020.8 ± 887.9	F(2, 46) = 2.72; *p* = 0.07

Note: One patient was excluded in NPPE group (*n* = 14) due to different acquisition parameters for the analysis

Post-hoc analysis for caudal anterior cingulate area showed that the significant differences were between HC and PPE (*p* = 0.042); for the left parahippocampal area, significant differences were evident between HC vs NPPE (*p* = 0.014) and NPPE vs PPE (*p* = 0.040); for the left superior temporal and for postcentral area there were no significant differences between groups

## Discussion

This is the first study examining brain structure in women at risk of developing PP. Our main finding is that women who developed a recent PP episode, compared with at risk women who did not develop a PP episode, have a reduction in volume of parahippocampal gyrus, ACC, postcentral gyrus and STG. These areas are implicated in the pathophysiology of psychosis, and these alterations may indicate a general vulnerability to developing these symptoms. Remarkably, our results revealed that differences in cortical volume in these regions might be driven by surface area changes rather than changes in cortical thickness. In addition, we found that women at risk who do not develop PP episodes have larger volumes of superior and inferior frontal gyri when compared with HC, suggesting a different cortical phenotype across the three groups.

Our finding of a smaller parahippocampal gyrus in women with PPE is consistent with evidence from studies in individuals with other psychoses^40–42^, and most interestingly in individuals at risk of psychosis^43–45^ who go on to develop psychosis (unrelated to the puerperium). This volumetric reduction may represent a predictive marker of illness progression. Our data also show that the STG is smaller in women with PPE compared to those at risk who do not suffer NPPE. This region contains several important structures, including the primary auditory cortex^46^, an area relevant to language, that has been described to contribute to the development of hallucinations^47, 48^. Our results show a smaller ACC in women with PPE compared to those with NPPE. This is consistent with findings from other groups with, or at risk of, affective psychosis^7,16,18, 49^. Remarkably, the reduction we observed was centered in the dorsal part of the ACC, rather than in the subgenual area, as previously reported for affective disorders^7,23,49,50,]^. Nevertheless, smaller volumes of dorsal and posterior ACC^51^ have been associated with psychotic disorders^52^ and poorer clinical outcome and social functioning in patients with bipolar disorder^52, 53^.

Women at risk of PP who did not develop PPE showed larger superior frontal gyrus and inferior frontal gyrus than HC and of PPE albeit at a trend level. Abnormalities in frontal regions are consistent with evidence of morphological changes in these areas in psychosis. Still, frontal regions have more often been reported to be smaller^23, 43^, rather than larger^53^, in individuals at risk of psychosis and with established psychosis not related to the puerperium ^23,46, 54–56^. Although our findings confirm that these could be areas of vulnerability, the difference in direction raises the issue as to whether these plastic changes occur specifically in the perinatal period, in relation to delivery, hydration, illness stage or medication usage. To this end, Kim et al.^57^ recently studied healthy women in the early postpartum period and found larger gray matter volume in the prefrontal cortex, parietal lobe and midbrain areas, suggesting that the first months of motherhood are accompanied by significant changes in brain structure. It may be that those changes in women at risk of psychosis reflect a protective effect or a reduced vulnerability to PP episodes.

Longitudinal studies have shown that patients with affective psychosis, but not those with schizophrenia, show enlargement of frontal and temporal areas after the first psychotic episode^58^. This, however, might reflect a medication-driven effect, not necessarily associated with a specific clinical phenotype^59^. Interestingly, our at risk NPPE group included a large number of women with bipolar disorder (67% vs 18%), but there were no differences in antipsychotic dose between the NPPE and PPE groups and only one participant was on lithium at the time of the MRI (Table 1). Nonetheless, it is worth noting that the NPPE group had been exposed to medication for longer than the PPE, possibly because of their longer duration of illness.

We also found smaller postcentral gyrus volume in women with PPE relative to NPPE although only at trend level. Indeed, parietal areas have been reported to be smaller in patients at the early stages of a psychotic illness^60^ and also in individuals at risk who later develop psychosis^14,16, 42^.

Finally, when we evaluated CTh and CSA in the regions that differed between groups, we found that between-group differences in ACC and parahippocampal gyrus were driven by reductions in CSA and not in CTh, in line with the stronger relationship previously reported between volume and CSA in healthy volunteers^61^. CTh growth and CSA expansion result from increases in dendritic arborization, axonal elongation, thickening, synaptogenesis and glial proliferation. These two measures are both highly heritable but not collinear^62, 63^. While thickness growth is complete at 2 years of age, CSA expansion continues into adulthood, with the addition of minicolumns within the cortical surface^64, 65^. Recent evidence shows reductions in frontal areas in cortical thickness, area and volume in patients with bipolar disorder in comparison to HC^13^. However, Rimol et al.^66^ have suggested that cortical structural differences between patients with schizophrenia and those with bipolar disorder may be driven by CSA, while differences between patients with schizophrenia and HC are mostly related to CTh. It is possible that differences in findings are related to the population characteristics, such as medication exposure or age at onset. For example, a study in patients with adolescent-onset (rather than adult) schizophrenia found that the smaller volumes shown in frontal and temporal areas in these patients when compared to controls were mostly attributable to smaller local surface area^67^. In our study, we found smaller parahippocampal and STG CSA (although STG did not survive post-hoc comparison) in the PPE group than in the NPPE group, which had a greater proportion of non-affective psychosis. The fact that CTh and CSA may represent disorder- and time-specific trajectories suggests that genetic, environmental and experience-dependent processes may contribute to the differential development of these cortical volume components. CTh and CSA should therefore be evaluated separately, as they can provide further insight into the neurobiological mechanisms associated with brain structural changes.

Our study provides novel findings for PP research. Women at risk of PP constitute a valuable model of vulnerability to psychosis, since the population is homogeneous in terms of gender, risk and illness onset, which is related to a unique biological event, childbirth. Therefore, comparing women at risk who do not develop psychosis and women at risk who do allows the identification of markers specific to the lifetime occurrence of PP. Moreover, using an HC group in the same postpartum period is crucial to reduce the likelihood of identifying differences that could be due to hormonal or hemodynamic changes.

There are important limitations that need to be acknowledged. Although this is the first neuroimaging study of women at risk of PP, the sample size is relatively small. In addition, the women in our cohort were not drug naïve, with 76% taking antipsychotic treatment at the time of MRI. This is an important factor to consider, since exposure to antipsychotic drugs has been found to be associated with changes in brain volumes in both cortical and subcortical areas^68^. Unfortunately, we were not able to fully explore the potential role of medications as we did not have full details of previous pharmacological treatment and length of exposure. Nevertheless, when we controlled for daily antipsychotic dosage this did not change the results, suggesting that an effect of medications is unlikely to fully explain the differences we identified. Another factor that has been associated with cortical volume in psychosis studies is duration of illness^69^. In our sample, duration of illness was shorter in women in the PPE group. Therefore, we explored whether duration of illness was associated with the volumes of the regions we evaluated, but found no correlation with any of the areas (supplementary Table 1). Similarly, in women with PPE there was a longer interval between delivery and acquisition of the MRI, and we cannot exclude that this could have played a role in the morphological differences we observed, as it is unclear for how long any brain change observed may last after delivery^70^. Future work should include longitudinal studies to measure the trajectory of brain structural changes associated with psychosis and motherhood.

In conclusion, we provide first, preliminary evidence that reductions in superior and medial temporal regions and in the anterior cingulate could represent risk markers of vulnerability to PP episodes in women at risk. These findings should be validated in larger samples of women at risk, as the presence of these markers could potentially help identify those women at risk who are more likely to develop an episode of illness in the postpartum period.

## Electronic supplementary material

Supplementary Table 1
