# Supplementary material for: Brain structure in women at risk of postpartum psychosis: an MRI study
Source: Transl Psychiatry. 2017 Dec 18;7:1286. doi: 10.1038/s41398-017-0003-8 (PMC5802701; doi:10.1038/s41398-017-0003-8)
Supplement: Supplementary file 1 — Supplementary Table 1 [file 41398_2017_3_MOESM1_ESM.docx]

**Supplementary Table 1.** Partial correlations between the volume of the regions of interest and number of weeks after delivery and length of illness (including age as a covariate of no interest).

| **Cortical Region** | **Partial correlation**  **(covarying for age)** | **Partial correlation**  **(covarying for age)** |  |  |
| --- | --- | --- | --- | --- |
|  | **Weeks after delivery (time to scan)** | **Length of Illness** |  |  |
| **Anterior Cingulate Cortex** | -0.23, p=0.1 | 0.04, p=0.8 |  |  |
| **Left Parahippocampal gyrus** | -0.07, p=0.6 | -0.03, p=0.8 |  |  |
| **Left Superior temporal Gyrus** | -0.164, p=0.2 | 0.036, p=0.8 |  |  |
| **Left Superior Frontal Gyrus** | 0.183, p=0.2 | 0.09, p=0.5 |  |  |
| **Left Inferior Frontal Gyrus** | 0.03, p=0.8 | 0.06, p=0.7 |  |  |
